# Abcès de la face révélant une sinusite maxillaire d'origine dentaire

**DOI:** 10.11604/pamj.2019.32.115.14103

**Published:** 2019-03-12

**Authors:** Souha Kallel, Abdel Mounem Ghorbel

**Affiliations:** 1 Service ORL et Chirurgie Cervico-faciale, CHU Habib Bourguiba, 3029 Sfax, Tunisie

**Keywords:** Sinusite maxillaire, origine dentaire, abcès facial, Maxillary sinusitis, dental origin, facial abscess

## Abstract

Maxillary sinusitis is exceptionally externalized. We report an unusual case of maxillary sinusitis externalized through the average meatus by following the intersinusonasal bulkhead toward the anterior angle of the maxilla and the nasogenian sulcus. The study involved a patient aged 17 years, with no previous medical history, presenting with facial swelling associated with right nasal obstruction occurred two days before. Clinical examination revealed febrile patient with abscess in the right nasogenian sulcus (A), with total filling of the right nasal fossa by a renitent submucosal bulge. Contrast-enhanced CT scan of the facial bones showed right maxillary sinusitis enlarging the average meatus (B) with collections in the intersinusonasal bulkhead and in the nasogenian sulcus. Furthermore, it showed intrasinusal migration of the 17th tooth root (C). The diagnosis of maxillary sinusitis of dental origin exteriorized to the face was made. Under general anesthesia, drainage of the different purulent collections through an incision in the nasal vestibule, middle meatotomy for drainage of the maxillary sinus and extraction of the 17^th^ tooth were performed. Patient's evolution was favorable with regression of fever and of inflammatory signs.

## Image en médecine

La sinusite maxillaire s'extériorise exceptionnellement. Nous rapportons une voie inhabituelle d'extériorisation à travers le méat moyen en suivant la cloison intersinuso-nasale vers l'angle antérieur de l'os maxillaire et le sillon naso-génien. Il s'agissait d'un patient âgé de 17 ans, sans antécédents particuliers, qui a consulté pour l'apparition depuis deux jours d'une tuméfaction de la face avec une obstruction nasale droite. A l'examen, il était fébrile avec présence d'une tuméfaction inflammatoire collectée du sillon naso-génien droit (A) avec comblement total de la fosse nasale droite par un bombement sous muqueux rénitent. Un scanner injecté du massif facial a montré une sinusite maxillaire droite élargissant le méat moyen (B) avec des collections au niveau de la cloison intersinuso-nasale et du sillon nasogénien. Par ailleurs, on visualise une migration de la 17^ème^racine dentaire en intra-sinusien (C). D'où le diagnostic d'une sinusite maxillaire d'origine dentaire extériorisée à la face a été déduit. Sous anesthésie générale, le patient a eu un drainage des différentes collections purulentes à travers une incision du vestibule nasal, une méatotomie moyenne pour drainage du sinus maxillaire et une extraction de la 17^ème^ dent. L'évolution était favorable avec régression de la fièvre et des signes inflammatoires.

**Figure 1:**
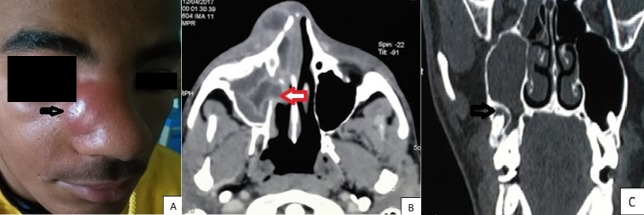
A) abcès comblant la région nasogénienne droite de la face; B) TDM du massif facial en coupe axiale; C) TDM du massif facial en coupe coronale

